# Sex-specific hypothalamic PVN transcriptomic signatures of blood pressure autonomic regulation and neuroinflammation in hypertension

**DOI:** 10.1186/s13293-026-00855-3

**Published:** 2026-02-21

**Authors:** V. J. Duque, J. V. Nani, M. Jovanovic, M. Lozić, O. Šarenac, A. G. Pauža, D. M. Murphy, N. Z. Japundžić-Žigon, A. S. Mecawi

**Affiliations:** 1 Laboratory of Molecular Neuroendocrinology, Department of Biophysics, Paulista School of Medicine, Federal University of São Paulo, São Paulo, Brazil; 2 Department of Pathophysiology, University of Belgrade Faculty of Medicine, Belgrade, RS Serbia; 3 Department of Pharmacology, University of Belgrade Faculty of Medicine, Belgrade, RS Serbia; 4 Department of Safety Pharmacology, AbbVie, North Chicago, Illinois USA; 5 Molecular Neuroendocrinology Research Group, Bristol Medical School: Translational Health Sciences, Dorothy Hodgkin Building, University of Bristol, Bristol, UK; 6 Manaaki Mānawa, The Centre for Heart Research, Department of Physiology, Faculty of Medical & Health Sciences, University of Auckland, Auckland, New Zealand

**Keywords:** Paraventricular nucleus, Blood pressure, Sex differences, Transcriptomics, Inflammation, Anti-inflammatory, Cardiovascular, Hypertension, RNA-Seq

## Abstract

**Introduction:**

Hypertension is a multifactorial condition of unknown cause that affects more than 1.28 billion adults worldwide and impacts the sexes differently. The hypothalamic paraventricular nucleus (PVN) plays a central role in blood pressure (BP) regulation by modulating sympathetic tone and releasing neuropeptides that affect the cardiovascular function. In this study, we investigated the transcriptomic profile of the PVN in hypertensive strains and across sexes, aiming to identify novel sex-specific molecular pathways involved in the regulation of BP.

**Methods:**

To accomplish this goal, we sequenced RNA from the PVNs of normotensive Wistar rats and Spontaneously Hypertensive Rats (SHR), both male and female. We also performed a cardiovascular assessment based on blood pressure (BP) measurements and their variability.

**Results:**

Cardiovascular assessment revealed higher SBP in SHRs than in Wistar rats; while males exhibited greater autonomic regulation associated with vasomotor and neurohumoral mechanisms, while females maintained comparable SBP levels primarily through an increase in heart rate, reflecting distinct autonomic adaptations. Hypertension also impacted gene expression, with influences from both the hypertensive state and sex. Compared with female SHRs, male SHRs presented a marked increase in differentially expressed genes (DEGs). Key upregulated genes in males, including *Brain-Derived Neurotrophic Factor (Bdnf)* and *Hypocretin (Hcrt)*, have already been linked to elevated BP, and *Angiotensin II Receptor Type 1 (Agtr1a)* is possibly associated with increased SBP-VLF variability, which serves as an indirect measure of enhanced sympathetic tone. In contrast, the female transcriptomic signature was characterized by the upregulation of anti-inflammatory pathways, with upregulation of *NLR Family CARD Domain Containing 3 (Nlrc3)* and *Paired Ig-like Receptor B (Pirb)*, and downregulation of *Absent in Melanoma 2 (Aim2)*, and *S100 Calcium Binding Protein B (S100b).* Notably, genes associated with neuroinflammation, such as the downregulation of *Annexin A1 (Anxa1)* and the upregulation of *Solute Carrier Family 11 Member 1 (Slc11a1)*, were consistently altered in both sexes.

**Conclusion:**

These results provide new insights into the cardiovascular and molecular basis of sex differences in hypertension, suggesting distinct neurohumoral autonomic profile in males, whereas in females a greater anti-inflammatory component. These findings offer a valuable framework for developing future sex-specific therapeutic strategies.

**Supplementary Information:**

The online version contains supplementary material available at 10.1186/s13293-026-00855-3.

## Abstract (plain language summary)

Hypertension is a complex condition of unknown cause that affects more than 1.28 billion adults worldwide and impacts the sexes differently. The hypothalamic paraventricular nucleus plays a central role in blood pressure regulation by modulating sympathetic tone and releasing neuropeptides that affect blood pressure. In this study, we investigated the gene expression profile of the PVN in male and female Wistar rats and in Spontaneously Hypertensive Rats to identify the mechanisms involved in the regulation of blood pressure. RNA-sequencing analysis revealed that distinct gene expression profiles were influenced by sex and hypertensive conditions. We demonstrated that hypertensive male rats presented an increased number of differentially expressed genes, indicating a greater impact of hypertension on the paraventricular nucleus in males. The key upregulated genes in males include those previously linked in the literature to elevated blood pressure. In contrast, the female transcriptome was characterized by the upregulation of anti-inflammatory genes, which may mitigate brain inflammation in the paraventricular nucleus, potentially offsetting hypertensive mechanisms. Notably, genes associated with neuroinflammation were identified and found to be conserved between sexes. In conclusion, our data provide new insights into the key mechanisms underlying hypertension, as well as the sex differences of this condition. We demonstrated anti-inflammatory effects in females, which may help explain the lower prevalence of hypertension during the premenopausal period. These results offer a valuable framework for understanding the pathophysiology of hypertension and for developing future sex-specific therapeutic strategies.

**Supplementary Information:**

The online version contains supplementary material available at 10.1186/s13293-026-00855-3.

## Highlights

- Cardiovascular assessment confirmed elevated blood pressure in SHRs compared with Wistar rats, with male SHRs exhibiting a more pronounced vasomotor/neurohumoral autonomic profile than females, whereas females demonstrated a higher heart rate.
- Transcriptomic profiling of the PVN revealed a greater number of differentially expressed genes in male SHRs, underscoring the impact of hypertension in males.
- Consistent downregulation of Anxa1 and upregulation of Slc11a1 across both sexes highlights a potential shared mechanism of neuroinflammation in hypertensive states.
- Key upregulated genes in male SHRs, including Bdnf and Agtr1a, may increases the baseline BP and enhance the VLF component of BP variability as a central feature of the male transcriptomic profile.
- Compared with male SHRs, female SHRs exhibited an anti-inflammatory signature characterized by upregulation of Nlrc3 and Pirb and downregulation of Aim2, and S100b, which contrasts with the pro-inflammatory state observed in males.

**Supplementary Information:**

The online version contains supplementary material available at 10.1186/s13293-026-00855-3.

## Background

Primary hypertension, characterized by persistently high blood pressure (BP), is a global health crisis affecting over 1.44 billion adults aged 30–79 years worldwide [1]. It is often known as a “silent killer” because of its asymptomatic nature and late diagnosis, leading to cardiovascular, cerebrovascular, and renal complications if left untreated [2–4]. To prevent these complications, adequate and timely reduction of BP is recommended [5]. Current pharmacotherapies have limited efficacy [6] and often require polypharmacy, especially in advanced stages of hypertension. Many genetic and environmental factors predispose individuals to the development of primary hypertension, but the fundamental causes remain elusive.

Central to the neuroendocrine and autonomic control of circulation is the paraventricular nucleus of the hypothalamus (PVN) [7, 8]. Its contribution to primary hypertension is well documented [9, 10], operating through at least three pathways [11]: magnocellular neurons (MCNs) of the PVN synthesize and secrete the neuropeptide hormone arginine vasopressin (AVP) into the circulation, where it increases BP by inducing vasoconstriction and regulating water balance, as confirmed in animal models [12–15] and humans [16]; the modulation of sympathetic outflow by presympathetic parvocellular neurons (PCNs). These neurons provide both direct projections to the intermediolateral cell column (IML), forming synapses with preganglionic neurons, and indirect projections to the rostral ventrolateral medulla (RVLM), thereby enhancing sympathetic drive to the periphery [9, 17]; and the regulation of the stress response [9] through corticotrophin-releasing hormone (CRH), which defines susceptibility to stress and predisposes individuals to hypertension [18, 19]. While under baseline physiological conditions, the PVN presympathetic PCNs are quiescent [9], they become activated in hypertension through multiple mechanisms, including salt load; the mineralocorticoid receptor MR; angiotensin AT1R; V1aR; insulin; leptin; the downregulation of glutamate uptake by astrocytes; and the production of proinflammatory cytokines [10, 13].

Significant sex differences exist in the prevalence and progression of the cardiovascular diseases (CVD) and hypertension. Among these differences, women are at greater risk for certain complications such as heart failure with preserved ejection fraction, atrial fibrillation, and stroke, and evidences suggests that these risks emerge at lower BP levels than in men, fueling the debate for sex-specific hypertension thresholds; in contrast, men are more prone to myocardial infarction and heart failure with reduced ejection fraction [20, 21]. BP differences are also evident, with premenopausal women generally exhibiting a protective phenotype [22] compared with men [23]. Although the peripheral vasodilatory and BP protective effects of sex hormones, especially estrogen, are well documented [22], the central neural mechanisms mediating these differences are still not fully elucidated. Sex hormone-dependent mechanisms have been discovered in the PVN that regulate AVP synthesis and release under basal conditions and stress. In the magnocellular part of the PVN, MCNs express the oestrogen receptor ERβ [24], and oestrogen exposure has been found to lower the plasma osmotic threshold for AVP release [25] as well as the threshold for the osmotic sensation of thirst [26]. Indeed, estrogen and progesterone exposure are associated with plasma volume expansion and a leftward shift in the osmotic operating point for body fluid regulation [27, 28]. In the parvocellular part of the PVN, PCN also expresses ERα and ERβ and membrane-bound ER, which increases AVP and CRH synthesis [29, 30]. Numerous studies point to sex differences in the hypothalamic‒pituitary axis (HPA) and show that neurochemical reactivity to stress is greater in females than in males [31]. Although many of the differences in BP regulation between sexes can be attributed to sex hormones, available clinical studies indicate that hormone replacement therapy in hypertensive postmenopausal women does not restore normal BP [32] suggesting a limited role of sex hormones and related mechanisms.

Given that the importance of the PVN as an integrative site for neuroendocrine and autonomic cardiovascular homeostasis is indisputable and that its contribution to the initiation and maintenance of hypertension is complex and multifactorial, we hypothesized that the PVN transcriptome may exhibit informative sexual dimorphism in the context of hypertension. We expected that spontaneously hypertensive (SHR) males would present a transcriptomic signature reflecting differential autonomic regulation and neuroinflammation, whereas SHR females would display a distinct, potentially protective, immunomodulatory profile. To test this hypothesis, we performed deep RNA sequencing of the PVN from hypertensive male and female rats and their normotensive counterparts, alongside a detailed cardiovascular autonomic assessment.

## Methods

### Animal procedures

All experimental procedures in this study adhered to the guidelines set forth by Directive 2010/63/EU of the European Parliament concerning the protection of animals used for scientific purposes, as well as the UK Animals (Scientific Procedures) Act 1986. The experimental protocol was approved by the Ethics Review Board of the School of Medicine, University of Belgrade, and the relevant ministry of the Republic of Serbia (licence no. 119–01–13/17/2015–09). Furthermore, all studies involving animals were conducted in compliance with the ARRIVE guidelines for reporting animal experiments [33, 34].

Male and female Wistar rats and SHR, aged 12 weeks (280–330 g), were obtained from the animal facility of Medical faculty University of Belgrade. Wistar rats were used as normotensive controls because they present lower BP and better-preserved cardiac function compared to other common normotensive strains, such as Wistar Kyoto rats [35]. Wistar and SHR strain were housed in a controlled environment (12 h/12 h light‒dark cycle, temperature 21 ± 2 °C and humidity 65 ± 9%) with free access to standard food pellets (0.2% w/v sodium content purchased from Veterinarski Zavod, Subotica, Republic of Serbia) and tap water. The sample size per experimental group was determined using ‘power sample size calculation’ software freely available at http://biostat.mc.vanderbilt.edu/twiki/bin/view/Main/ PowerSampleSize to achieve a power of 90% and a type I error probability of 0.05 on the basis of expected effect sizes from prior studies.

### Radiotelemetry implantation and hemodynamic studies

All animals were subjected to the same surgical procedure and perioperative care. The animals were anesthetized via a combination of ketamine (100 mg•kg − 1, I.M.) and xylazine (10 mg•kg − 1, I.M.). Rat body temperature was maintained by a heating pad (Harvard Apparatus, Holliston, MA, USA). Following medial abdominal 3 cm-long incision and intestine retraction, a catheter tip of the radiotelemetry device (TA11-PA C40; DSI, Transoma Medical, St Paul, MN, USA) was inserted into the aorta and fixed with 3 M VetbondTM and a tissue cellulose patch (DSI, Transoma Medical). Rats were treated with gentamicin (25 mg•kg − 1, i.m.) and carprofen (5 mg•kg − 1 daily, s.c.) perioperatively for prevention of bacterial infection and pain relief, respectively. Postoperatively, the animals were housed individually (30 × 30 × 30 cm Plexiglas cage) under controlled laboratory conditions. They were monitored every day until they fully recovered.

After a two-week recovery period, now 14-weeks old rats, were submitted to one-hour long continuous BP recording between 10 a.m. and 11 a.m. in a separate room and quiet surrounding, a duration sufficient to capture a stable representation of autonomic tone, in four groups of animals: Wistar male rats (*n* = 6), Wistar female rats (*n* = 5, telemetric probe stopped working in one of the Wistar females), SHR males (*n* = 6), and SHR females (*n* = 6). Data acquisition from females was conducted during the dioestrus phase of the estrous cycle, as confirmed by daily examination of vaginal smears [36]. At the end of the registration period rats (14 weeks old) were euthanized by decapitation using Guillotine preceded by stunning, and brains were collected and stored at − 80 °C.

### Cardiovascular signal processing and analysis

Arterial BP was digitalized at 1000 Hz in Dataquest A.R.T. 4.0 software (DSI, Transoma Medical). Systolic (SBP) and diastolic BP (DBP) and pulse interval (PI) or its inverse heart rate (HR) was derived from the arterial pulse pressure wave as the maximum, minimum and dP/dt_max_ inter-distance, respectively. Spectral analysis of SBP, DBP and HR via fast Fourier transformation was performed after linear detrending, nine-point Hanning window filtering and resampling at 20 Hz [37]. Linear detrending was applied to prevent superposition of long-term BP oscillations (ultradian, circadian) to short-term oscillations. Spectra were obtained from 30 overlapping 2048-point time series (~ 7 min-long recordings) of SBP, DBP and HR. The BP (mmHg2) and HR (bpm2) spectral powers were calculated for the whole spectrum (0.0195–3 Hz) and in three frequency ranges: very low frequency (VLF, 0.0195–0.195 Hz), low frequency (LF, 0.195–0.8 Hz) and high frequency (HF, 0.8–3 Hz). LF-BP and LF/HF-HR are established indicators of sympathetic outflow to arterial blood vessels and the sympathovagal balance of the heart, as observed in both experimental studies [38] and clinical practice [39, 40].

### Evaluation of the spontaneous baroreceptor reflex via the sequence method

The method is explained in detail elsewhere [41]. A stream of at least four consecutively increasing or decreasing SBP values and PI values delayed by three, four or five beats with respect to SBP were considered baroreceptor reflex sequences [42]. The baroreceptor reflex sensitivity (BRS, ms•mmHg − 1) was calculated as a linear regression coefficient averaged over all identified sequences (PI = BRS•SBP + const., where fitting of the curve was performed via the least squares method).

### Statistical analysis (Hemodynamic data)

Hemodynamic data are presented as mean ± s.e.m. Differences between experimental groups were analyzed using GraphPad Prism software version 8.4.2 (GraphPad Software Inc., San Diego, CA, USA). Each group was first tested for normal distribution by the Shapiro-Wilk test. Cardiovascular parameters were then compared using two-factor ANOVA to evaluate the main effects of strain, sex, and their interaction followed by a post hoc (Tuckey test). Unpaired observations were additionally analyzed by T test for unpaired observations. A p-value < 0.05 was considered statistically significant.

### PVN sampling

Using a cryostat (Leica Microsystems CM1900, Leica Microsystems, Nussloch GmbH, Nussloch, Germany), we cut 60 μm-thick brain sections. The initial sections were stained with toluidine blue (1% [v/v] in 70% [v/v] ethanol, Sigma‒Aldrich Co. Ltd., Poole, Dorset, UK) to facilitate anatomical identification and localization of the hypothalamic PVN. Once the PVN was visually identified, we ceased staining and, from that point onward, bilateral tissue punches (1 mm diameter) were collected from the corresponding unstained sections using a 15-G microsampling needle (Sample Corer, Fine Science Tools Inc., Foster City, CA, USA; cat. no. 18035-01). The collected PVN samples were immediately stored in RNAse-free tubes (ISOLAB Laborgeräte GmbH, Eschau, Germany) at −80 °C.

### RNA extraction and sequencing

RNA extraction and library construction were performed as described previously [43]. Four groups of animals were used: Wistar male rats (*n* = 4), Wistar female rats (*n* = 5), SHR males (*n* = 5), and SHR females (*n* = 5). The samples were lysed via the QIAzol lysis reagent (Qiagen), and total RNA was extracted via the Direct-zol RNA MiniPrep extraction kit (Zymo Research, Irvine, CA, USA). The RNA integrity number (RIN) was determined via an Agilent 2200 TapeStation system (Agilent Technologies, 2503). Samples with a RIN > 8 were used for sequencing. cDNA libraries were prepared via the TruSeq^®^ Stranded mRNA Library Prep (Illumina, 20020594) and sequenced via the NextSeq500 High Output Version 2.5, 2 × 75 bp kit (Illumina, 15057931) at Bristol Genomics Facility, University of Bristol. Each sample generated > 35 million paired-end reads, according to MultiQC [44].

### RNA-seq data analysis

Paired-end sequencing data (FASTQ) generated on the Illumina platform were first processed via a trimming step (GSE:309435). This preprocessing included adaptor clipping, quality-based filtering, and length selection, carried out with Trimmomatic [45], using a Phred score threshold of 30 (≈ 99.9% base call accuracy). Post-trimming quality assessment was performed with MultiQC [44], and the resulting high-quality reads were then aligned to the *Rattus norvegicus* reference genome (Rn7) using the Spliced Transcripts Alignment to a Reference (STAR) aligner [46]. The counts of aligned reads were calculated using FeatureCountsvia featureCounts [47].

Statistical analyses were conducted in the R programming language (version 4.4.1) [48]. The data were first normalized via the median-of-ratios method [49] built into DESeq2 (version 1.44.0) [50]. Differentially expressed genes (DEGs) were identified using the Wald test implemented in DESeq2, with p-values corrected using the Bonferroni method. DEGs with adjusted p-values (padj) < 0.05 were considered statistically significant. Gene annotation was performed via the ClusterProfiler package (version 4.10.1) [51], which retrieves genome annotations from ENSEMBL [52]. For correlation and linear regression analyses, we applied the Spearman method from the Hmisc package (version 5.1–3) and the Stats package (version 4.3.1), respectively. The data were visualized via custom scripts written in R via the “ggplot2” (v3.5) package.

### Functional classification and gene ontology

To explore the functional profiles of the DEGs, we organized the genes according to their physiological and pharmacological roles. Gene classification was carried out following the categories proposed by The International Union of Basic and Clinical Pharmacology (IUPHAR) system [53], which groups genes into categories such as endogenous peptides, G protein-coupled receptors, catalytic receptors, enzymes, channels, transporters, and other pharmacological targets. In addition, we defined an extra category specifically for transcription factors that have been previously described in the human literature [54].

To investigate the pathways modulated by the DEGs, we performed functional enrichment analyses via Gene Ontology (GO) and Reactome, implemented through the Cluster Profiler (version 4.10.1) [51] and Reactome (version 1.48) [55] packages. These analyses identified pathways in the biological process (BP), molecular function (MF), and cellular component (CC) categories and the Kyoto Encyclopedia of Genes and Genomes (KEGG) and Reactome categories [55–57]. To use these tools, the first step was to convert the gene symbols to Entrez IDs via the ClusterProfiler package [51], which uses only genes with a BaseMean ≥ 5 as the background for overrepresentation analysis. The analyses were conducted with the org.Rn.eg.db database (version 3.18.0). Pathways were considered significantly altered when the P. Adjusted were < 0.05.

### Data integration

Aiming to improve the understanding of molecular differences in key brain regions regulating BP, we analyzed previously published RNA transcriptomic datasets comparing SHR and normotensive Kyoto rats (12 and 16 week-old females) from the Nucleus of the Solitary Tract (NTS), Caudal Ventrolateral Medulla (CVLM), and Rostral Ventrolateral Medulla (RVLM)[58], to identify statistically significant differences. The raw data were obtained from Gene Expression Omnibus (GSE234784), and the counts were normalized and analyzed following the same parameters as our original dataset. Since a list of differentially expressed genes was not available, we used the raw count data for our analysis.

## Results

### Hemodynamic parameters and autonomic portrayal of Wistar and SHR males and females

**Fig. 1:**
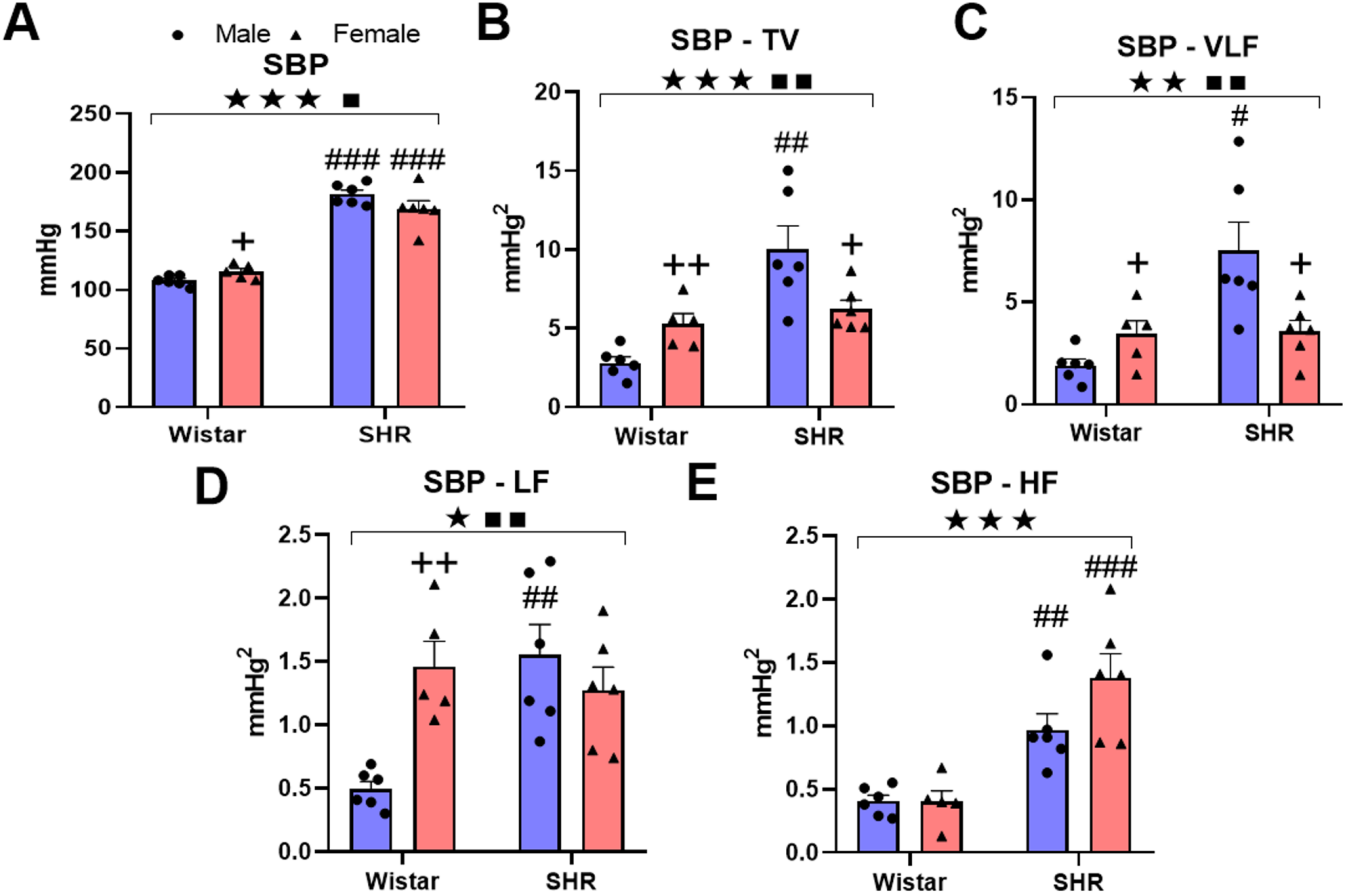
Systolic BP Levels and Variability. (**A**) Bar plot showing systolic blood pressure (SBP) values across sex and lineage, as well as short-term SBP variability in SHR and Wistar of both sexes. The blue bars indicate males, whereas the red bars indicate females. (**B–E**) Bar plots representing short-term variability in total variance (**B**), very-low-frequency (VLF) (**C**), low-frequency (LF) (**D**), and high-frequency (HF) (**E**) components. Note the effects of strain and the strain × sex interaction on SBP and SBP variability. ★ *p* < 0.05, ★★ *p* < 0.01, ★★★ *p* < 0.001: strain effect (comparison between SHR and Wistar rats within the same sex); ∎ *p* < 0.05, ∎∎ *p* < 0.01: strain × sex interaction (two-way ANOVA); + *p* < 0.05, ++ *p* < 0.01: female compared with male within the same strain; # *p* < 0.05, ## *p* < 0.01, ### *p* < 0.001: intergroup comparison (comparison between SHR and Wistar rats within the same sex; Tuckey post hoc)

Physiological analysis revealed significant differences in cardiovascular parameters between SHRs and Wistar, with distinct profiles for males and females. For SBP, two-way ANOVA revealed significant main effects of strain (F(1,19) = 215.509, *P* ≤ 0.0001) and a significant strain × sex interaction (F(1,19) = 5.385, *P* = 0.032) (Fig. 1A). This pattern extends to SBP variability, with significant strain effects and interactions for total variability (TV-SBP: F(1,19) = 20.346, *P* ≤ 0.0001; F(1,19) = 12.135, *P* = 0.002), very-low-frequency SBP (VLF-SBP: F(1,19) = 11.157, *P* = 0.003; F(1,19) = 10.132, *P* = 0.005) and low-frequency SBP (LF-SBP: F(1,19) = 5.5741, *P* = 0.029; F(1,19) = 11.458, *P* = 0.003) (Fig. 1B-D). High-frequency SBP (HF-SBP) analysis revealed only a strain effect (F(1,19) = 35.6, *P* ≤ 0.0001) (Fig. 1E). Post hoc tests confirmed that SBP was greater in SHRs than in Wistar in both sexes. Notably, SHR males presented significantly increased TV-SBP and other variability metrics compared with those of Wistar males, whereas SHR females differed from Wistar females only in HF-SBP. Within-strain comparisons revealed that SHR females indicates a less pronounced autonomic regulation associated with vasomotor and neurohumoral mechanisms, consistent with a less severe autonomic phenotype.

Similar patterns were observed for diastolic blood pressure (DBP), with significant strain and interaction effects on DBP (F(1,19) = 65.404, *P* ≤ 0.0001; F(1,19) = 5.275, *P* = 0.033), as well as in the variability analyses, with TV-DBP (F(1,19) = 4.79, *P* = 0.041; F(1,19) = 4.211, *P* = 0.05) and VLF-DBP (F(1,19) = 4.781 *P* = 0.041; F(1,19) = 4.052, *P* = 0.05) (Supplementary Fig. 1A-C). A strain × sex interaction effect on LF-DBP was also observed (F(1,19) = 7.650, *P* ≤ 0.012; F(1,19) = 6.693, *P* = 0.018) (Supplementary Fig. 1D). Strain alone influenced HF-DBP (F(1,19) = 16.603, *P* = 0.001) (Supplementary Fig. 1E). Intergroup comparisons revealed higher DBP, TV-DBP, VLF-DBP, LF-DBP and HF-DBP in SHR males than in Wistar males. In SHR females, DBP and HF-DBP were greater than they were in Wistar females. Intragroup differences indicate greater normal DBP, TV-DBP, and LF-DBP in Wistar females than in males, whereas no significant difference in DBP or its variability was detected between male and female SHRs.

We also analyzed heart rate (HR), which demonstrated a significant sex effect (F(1,19) = 9.611, *P* = 0.006) (Supplementary Fig. 2 A), where intergroup comparisons revealed higher HRs in SHRs than in males. Furthermore, in terms of variability analyses, we observed a strain effect on TV-HR (F(1,19) = 4.789, *P* = 0.041) (Supplementary Fig. 2B) and VLF-HR (F(1,19) = 5.219, *P* = 0.034) (Supplementary Fig. 2 C) and a sex effect on LF-HR (F(1,19) = 7.743, *P* = 0.012) (Supplementary Fig. 2D). For HF-HR, no significant effects of sex or strain were observed (Supplementary Fig. 2E). However, our analyses of the LF/HF HR ratio (Supplementary Fig. 2 F) revealed significant effects of both sex (F(1,19) = 6.912, *P* = 0.017) and strain (F(1,19) = 6.648, *P* = 0.018). Finally, the BRS analysis revealed no significant effects of strain, sex, or their interaction (F(1,19) = 2.630, *P* = 0.121; F(1,19) = 0.570, *P* = 0.460; F(1,19) = 0.052, *P* = 0.821; data not shown).

### Characterization of the PVN transcriptomic profile in both sexes

RNA sequencing of the PVN identified over 21,000 expressed genes, with a core set of ~ 17,400 genes with a mean expression greater than 5 retained for differential analysis. These genes are distributed across all chromosomes in males and females (Supplementary Fig. 3 A and B, Supplementary Table 1), including their median expression. Next, we categorized the genes based on IUPHAR and TF classification and selected those with the highest expression levels in both males and females. Our analysis revealed strong conservation of expressed genes between the sexes and several expected genes, such as those in the endogenous peptide classification, including Oxytocin (*Oxt*), Vasopressin (*Avp*), and Angiotensinogen (*Agt*), in male (Supplementary Fig. 3 C, Supplementary Table 2) and female rats (Supplementary Fig. 3D, Supplementary Table 2).

### Sex differences in the PVN transcriptome in normotensive and hypertensive rats

**Fig. 2:**
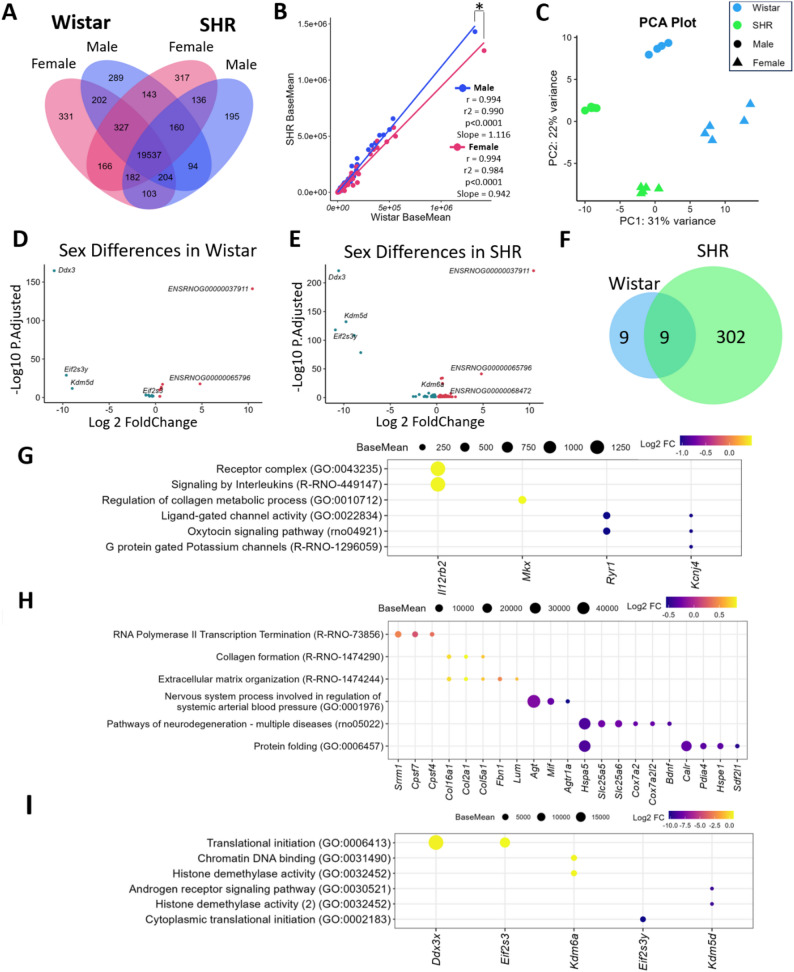
Sex Differences Across Strains: **(A)** Venn diagram showing the genes expressed in each sex and strain. **(B)** Scatter plot of gene expression; each dot represents a gene from Wistar or SHR rats. Spearman correlations and linear regressions for male and female rats are also shown. An asterisk (*) indicates a difference in slopes (*p* ≤ 0.001). **(C)** PCA demonstrating the clustering of samples according to sex and strain. (D-E) Volcano plots of sex-regulated genes in Wistar **(D)** and SHR **(E)** rats, with blue dots indicating downregulation and red indicating upregulation in females. **(F)** Venn diagram showing the coexpression of DEGs compared by sex in each strain. (G-I) Gene ontology analysis showing some of the significant pathways related to the unique genes upregulated and downregulated in the female compared with male in Wistar **(G)**, SHR **(H**) or both strains **(I)**. The five most differentially expressed genes in each pathway are highlighted, with point size denoting the base mean expression and color indicating the log2 fold change in females

We observed strong conservation among the genes identified and expressed in each sex and strain (Fig. 2A). Further analysis of the base mean counts of each gene in males and females revealed a strong correlation between Wistar and SHR (males: *r* = 0.994, slope = 1.116; females: *r* = 0.994, slope = 0.942). However, a significant difference in the regression slopes was observed (*p* < 0.0001; Fig. 2B). While both sexes presented highly correlated gene expression patterns between SHR and Wistar, the males presented a greater increase in gene expression in the SHR strain (slope above 1), with females showing a slight decrease in gene expression (slope below 1). This could indicate sex-specific differences in how gene expression is regulated between the two strains. (Fig. 2B).

To better understand the impact of sex on the gene expression profiles of the two strains, we conducted a differential expression analysis. Principal Component Analysis (PCA) revealed that the samples clustered distinctly based on both strain and sex, confirming the strong dimorphism of the PVN transcriptomic profile (Fig. 2C). Delving deeper into the impact of sex on the PVN transcriptome, we found a modest effect in normotensive rats, with only 18 DEGs between Wistar female and male rats (8 upregulated, 10 downregulated) (Fig. 2D, Supplementary Table 3). In contrast, the difference was amplified in the SHR strain, with 311 DEGs between SHR females and males (168 upregulated, 143 downregulated) (Fig. 2E, Supplementary Table 4). Among these DEGs, 9 were commonly regulated in both strains, primarily those related to sex chromosomes (Fig. 2F).

The 10 upregulated genes in the PVN of female Wistar rats revealed GO terms related to the receptor complex (GO:0043235), interleukins (GO:0070671, GO:0032729, GO:0032609, GO:0032649, rno04060, R-RNO-447115, and R-RNO-449147), and collagen processes (GO:0032967, GO:0010714, GO:0032965, GO:0010712, GO:0030199, GO:0032964, and GO:0032963). Conversely, pathways associated with the 8 downregulated genes in the PVN of Wistar females and consequently enriched in males were associated with potassium channels (GO:0005242, R-RNO-1296041, and R-RNO-1296059), ligand-gated channel activities (GO:0005217, GO:0015276, GO:0022834, GO:0099094, and GO:0099604), and the oxytocin signaling pathway (rno04921) (Fig. 2G, Supplementary Table 5).

In the SHR strain, pathways enriched with genes upregulated in females revealed mechanisms related to RNA polymerase II (R-RNO-73856), the extracellular matrix and collagen organization (GO:0031012, GO:0062023, GO:0030020, GO:0005201, R-RNO-1474244, GO:0005581, GO:0098644, GO:0005583, GO:0098643, R-RNO-8948216, R-RNO-1650814, and R-RNO-1474290). However, pathways related to genes downregulated in females and consequently upregulated in males included nervous system processes involved in the regulation of systemic arterial blood pressure (GO:0001976), neurodegeneration (rno05012) and protein folding (GO:0006457 and GO:0061077) (Fig. 2H, Supplementary Table 6).

The nine genes commonly regulated by sex in both strains were related to the sex chromosomes, with six genes associated with the X chromosome, including *DEAD-Box Helicase 3 X-Linked (Ddx3x)*, *Eukaryotic Translation Initiation Factor 2 Subunit Gamma (Eif2s3)*, *Lysine Demethylase 6 A (Kdm6a)*,* Polysaccharide Biosynthesis Domain Containing 1 (Pbdc1*), and the pseudogenes *ENSRNOG00000037911* and *ENSRNOG00000065796*. Moreover, three genes related to the Y chromosome were observed: *DEAD-Box Helicase 3 Y-Linked (Ddx3/Ddx3y/ENSRNOG00000057231)*,* Lysine Demethylase 5D (Kdm5d)*, and *Eukaryotic Translation Initiation Factor 2 Subunit 3*,* Structural Gene Y-Linked (Eif2s3y)*. These common genes demonstrated a conserved alteration influenced by sex, with those enriched in females related to translational initiation (R-RNO-72737, R-RNO-72649, R-RNO-72613, GO:0031369, GO:0005852, and GO:0003743), chromatin DNA binding (GO:0031490), and the histone complex (GO:0141052, GO:0035097, and GO:0032452). In contrast, those enriched in males also influenced pathways related to translational initiation (GO:0002183, GO:0006413, GO:0003743, GO:0008135, and GO:0090079), histone complexes (GO:0141052, GO:0032452, and R-RNO-3214842) and steroid hormone receptors (GO:0030521, GO:0060765, GO:0050681, GO:0030518, and GO:0033143) (Fig. 2I, Supplementary Table 7).

### Transcriptomic signature of hypertensive males highlights autonomic dysregulation and stress pathways

**Fig. 3:**
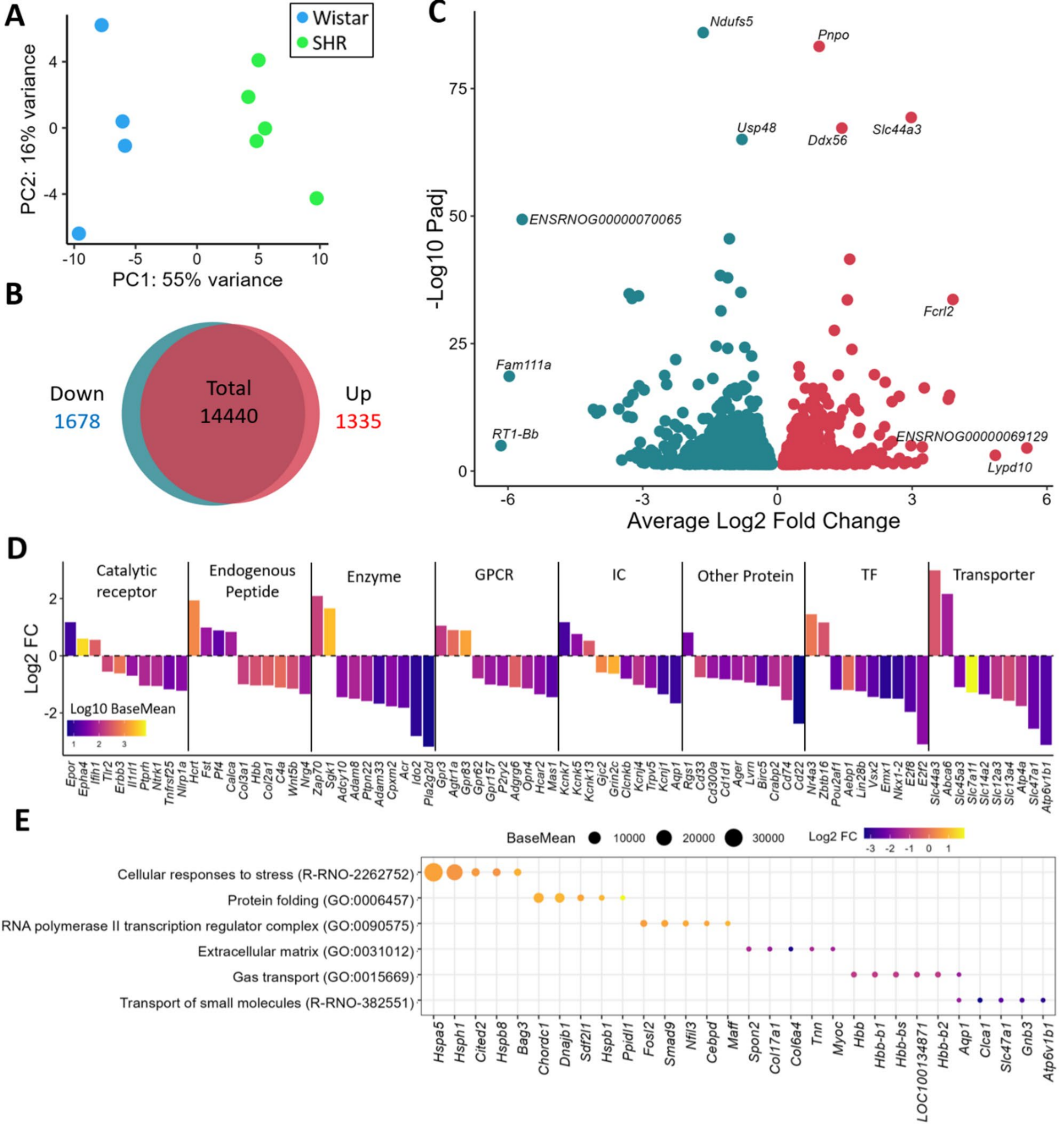
Transcriptomic Differences Between SHR and Wistar Males: **(A)** PCA plot showing the difference between Wistar and SHR male rats. Venn diagram **(B)** showing the number of genes downregulated and upregulated in SHR males compared with male Wistar rats, considering only genes with a mean expression higher than 5 in males. **(C)** Volcano plot showing the genes affected by the SHR, highlighting the top 3 downregulated and upregulated genes with the highest log2-fold change and most significant values. **(D)** IUPHAR classification of the top 10 genes in each IUPHAR + TF category, with size representing the log2-fold change and color representing the base mean in log10. **(E)** Gene ontology analysis showing some of the significant pathways related to genes whose expression was upregulated or downregulated in the SHR compared with Wistar in male rats. The top five most differentially expressed genes in each pathway are highlighted, with the size representing the base mean expression and the color indicating the log2-fold change (Male SHR compared with Male Wistar)

Transcriptomic differences between strains in male rats were clear in the PCA (Fig. 3A). A comparison of SHR males with Wistar males revealed a massive transcriptomic shift, with 3,013 DEGs (1,335 upregulated and 1,678 downregulated) (Fig. 3B, C and Supplementary Table 8). The categorization of these DEGs by the IUPHAR and TF databases revealed some potentially important genes for PVN functionality. The upregulated genes include *Brain-Derived Neurotrophic Factor (Bdnf)*, an endogenous peptide that serves as a key driver of BP regulation in the PVN. In the GPCR category, genes such as *Angiotensin II Receptor Type 1 (Agtr1a)*,* Atypical Chemokine Receptor 3 (Ackr3)*, and the catalytic receptor *TNF Receptor Superfamily Member 12 A (Tnfrsf12a)* were also upregulated (Fig. 3D, Supplementary Table 8). The differentially expressed genes also revealed important gene ontology terms, such as the upregulated genes, which were related to the cellular response to stress processes (R-RNO-2262752 and GO:0034976), protein folding (GO:0006457, GO:0051082, GO:0061077, GO:0051087, GO:0044183, GO:0140662, GO:0101031, GO:0006458, GO:0051084, GO:0051085, and GO:0042026), protein localization (GO:1903405, GO:1990173, GO:0034504, GO:1904851, GO:1900182, GO:0070203, GO:0070202, GO:0072594, GO:1900180, GO:1904816, and GO:0070200), and transcription complexes (GO:0090575). Conversely, the downregulated genes were associated with processes involving the extracellular matrix (GO:0031012, GO:0062023, GO:0030020, and GO:0005201) and gas transport (GO:0015669, GO:0005344, and GO:0015670) (Fig. 3E, Supplementary Table 9).

### Transcriptomic changes in the PVN of hypertensive female rats

**Fig. 4:**
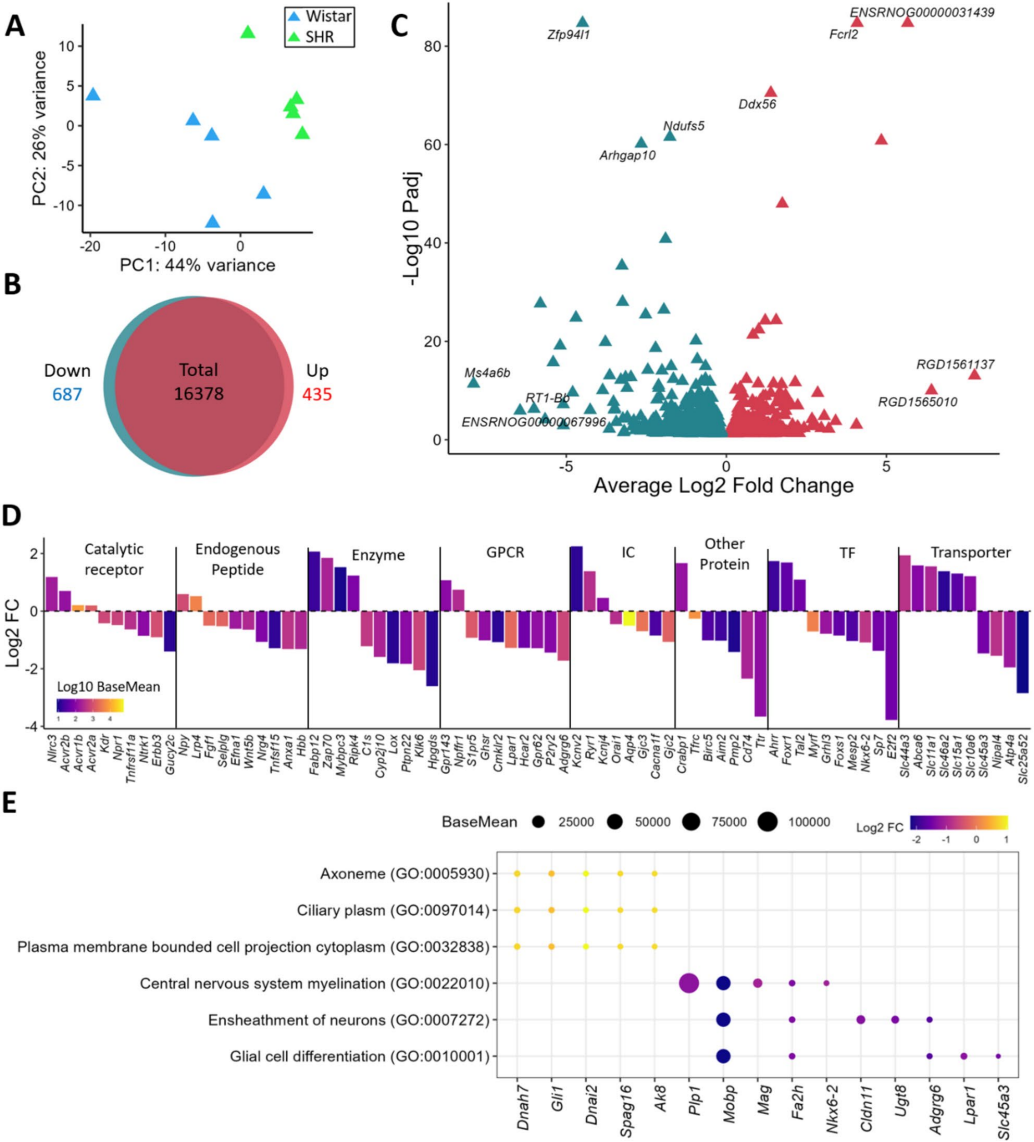
Transcriptomic Differences Between SHR and Wistar Females: **(A)** PCA plot showing the difference between Wistar and SHR female rats. Venn diagram **(B)** showing the number of genes downregulated and upregulated in SHR females compared with Wistar rats, considering only genes with a mean expression higher than 5 in males. **(C)** Volcano plot showing the genes affected by SHRs in female rats, highlighting the top 3 downregulated and upregulated genes with the highest log2-fold change and most significant values. **(D)** IUPHAR classification of the top 10 genes in each IUPHAR + TF category, with size representing the log2-fold change and color representing the base mean in log10. **(E)** Gene ontology analysis showing some of the significant pathways related to genes whose expression was upregulated or downregulated in the SHR compared with Wistar in female rats. The top five most differentially expressed genes in each pathway are highlighted, with the size representing the base mean expression and the color indicating the log2-fold change (Female SHR compared with Female Wistar)

The clear effect of strain on the PVN of female rats was also evident, as demonstrated by PCA (Fig. 4A). A comparison of SHR females with Wistar females revealed 1,122 DEGs (435 upregulated, 687 downregulated) (Fig. 4B, C and Supplementary Table 10). The categorization of these DEGs by the IUPHAR and TF databases revealed several potentially important genes that were differentially expressed in female SHRs compared with Wistars. Specifically, among the upregulated genes, the *NLR Family CARD Domain Containing 3 (Nlrc3)* was noted in the catalytic receptor category, whereas the downregulation of *Absent in Melanoma 2 (Aim2)* was observed in the other protein category (Fig. 4D, Supplementary Table 10). The impact of these transcriptomic alterations was also reflected in the gene ontology analyses, revealing pathways related to myelination (GO:0042552, GO:0022010, GO:0043209, GO:0043218, and GO:0019911), neuron ensheathment (GO:0032291, GO:0007272, and GO:0008366), and the regulation of glial cells (GO:0045685, GO:0010001, GO:0097386, GO:0021782, GO:0048713, GO:0014003, and GO:0048709) related to the downregulated genes. Moreover, pathways related to the axoneme (GO:0005930), ciliary plasm (GO:0097014), and plasma membrane-bound cell projection cytoplasm (GO:0032838) were enriched with the upregulated genes (Fig. 4E, Supplementary Table 11).

### Common and unique genes affected by hypertension in the PVN of male and female rats

**Fig. 5:**
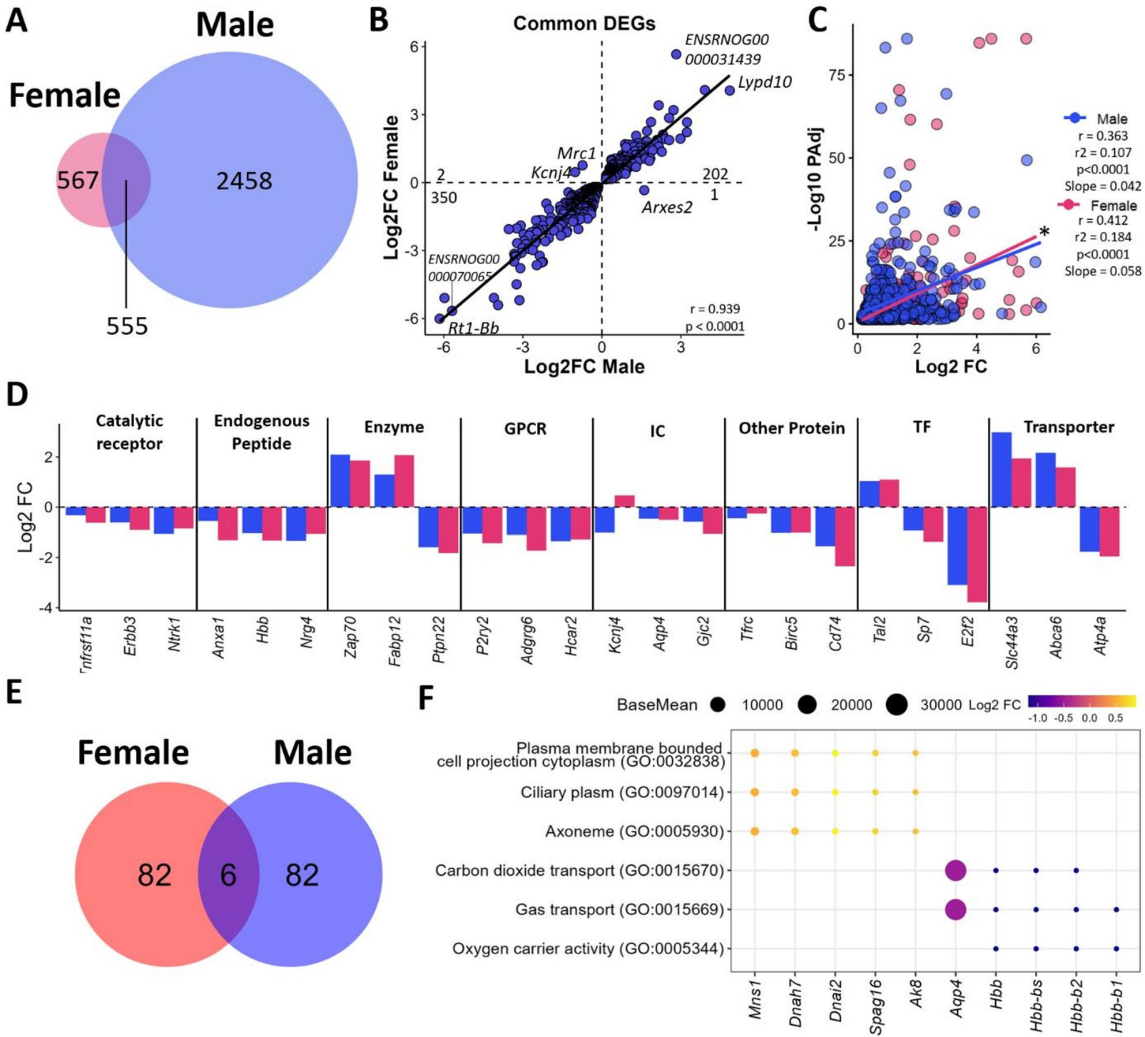
Shared DEGs Across Sexes: **(A)** Venn diagram showing the number of DEGs between Wistar and SHR rats in males and females. **(B)** Scatter plot showing the direction of change in common SHR-altered genes that are differentially regulated between sexes. **(C)** Scatter plot of common genes showing log₂ fold changes versus adjusted p-values for males and females. Spearman correlations and linear regression lines are displayed. * Indicates a significant difference between slopes (*p* ≤ 0.001). **(D)** Bar plot illustrating the top three common SHR-altered genes most affected within each TF and IUPHAR category, with expression levels shown for females (red) and males (blue). **(E)** Venn diagram representing the pathways (GO, KEGG and Reactome) differentially regulated in SHR strain (compared with Wistar) in male and female rats. **(F)** Dot plot demonstrating the commonly regulated pathways (GO, KEGG, and Reactome) in male and female rats of the SHR strain. The five most highly expressed genes in each pathway are highlighted, with point size representing the mean base expression in males and females, and color indicating the mean log₂ fold change (average of SHR versus Wistar values in males and females)

Compared with those in Wistars, the transcriptomic changes in the PVN of SHRs were notably more pronounced in males than in females. Among the DEGs, 2,458 DEGs were uniquely affected in males, and 567 were uniquely affected in females, according to the BP state of each sex, with 555 genes commonly affected in both sexes (Fig. 5A). A distribution analysis of the commonly regulated genes in both sexes revealed that 552 (99.45%) changed in the same direction and were significantly positively correlated between males and females (*R* = 0.942, *p* < 0.0001). Among these genes, several were upregulated in both males and females, such as *Serum/Glucocorticoid-Regulated Kinase 1 (Sgk1)* and *Ly6/PLAUR domain containing 10 (Lypd10)*, whereas *E2F Transcription Factor 2 (E2f2)* and *Amylase Alpha 1 A (Amy1)* were downregulated regardless of sex. Among the commonly regulated genes, three were significantly regulated in both sexes but in opposite directions: *Mannose Receptor C-Type 1 (Mrc1)* and *Potassium Inwardly Rectifying Channel Subfamily J Member 4 (Kcnj4)* were downregulated in males and upregulated in females, whereas *Adipocyte-related X-chromosome expressed sequence 2 (Arxes2)* was upregulated in males and downregulated in females (Fig. 5B, Supplementary Table 12). Furthermore, linear regression analysis comparing the effects of common genes on male and female DEGs (Fig. 5C) revealed that females presented a greater coefficient of determination (males: *r* = 0.363, R² = 0.107, slope = 0.042; females: *r* = 0.412, R² = 0.184, slope = 0.058), indicating a significant difference in slope (F = 17.17, *p* < 0.001) and suggesting a more coordinated transcriptomic response to hypertension in females than in males.

The classification of these common genes by the IUPHAR and TF databases revealed potentially important genes regulated by the SHR strain in each category. For example, in the enzyme category, there was an upregulation of *the zeta chain of T-cell receptor-associated protein kinase 70 (Zap70)* and *Sgk1*, whereas a downregulation of the endogenous peptide *Annexin A1 (Anxa1)* was observed (Fig. 5D, Supplementary Table 12). Among the pathways commonly affected by the hypertensive strain in male and female rats, six pathways were conserved (Fig. 5E). These included pathways related to cytoplasmic projection (GO:0032838), ciliary plasm (GO:0097014), and axoneme (GO:0005930) due to the positively regulated genes, whereas the negatively regulated genes affected gas transport pathways (GO:0015669, GO:0015670, and GO:0005344) (Fig. 5F, Supplementary Table 13).

### Associations between hypertension and sex differences in the PVN transcriptome

**Fig. 6:**
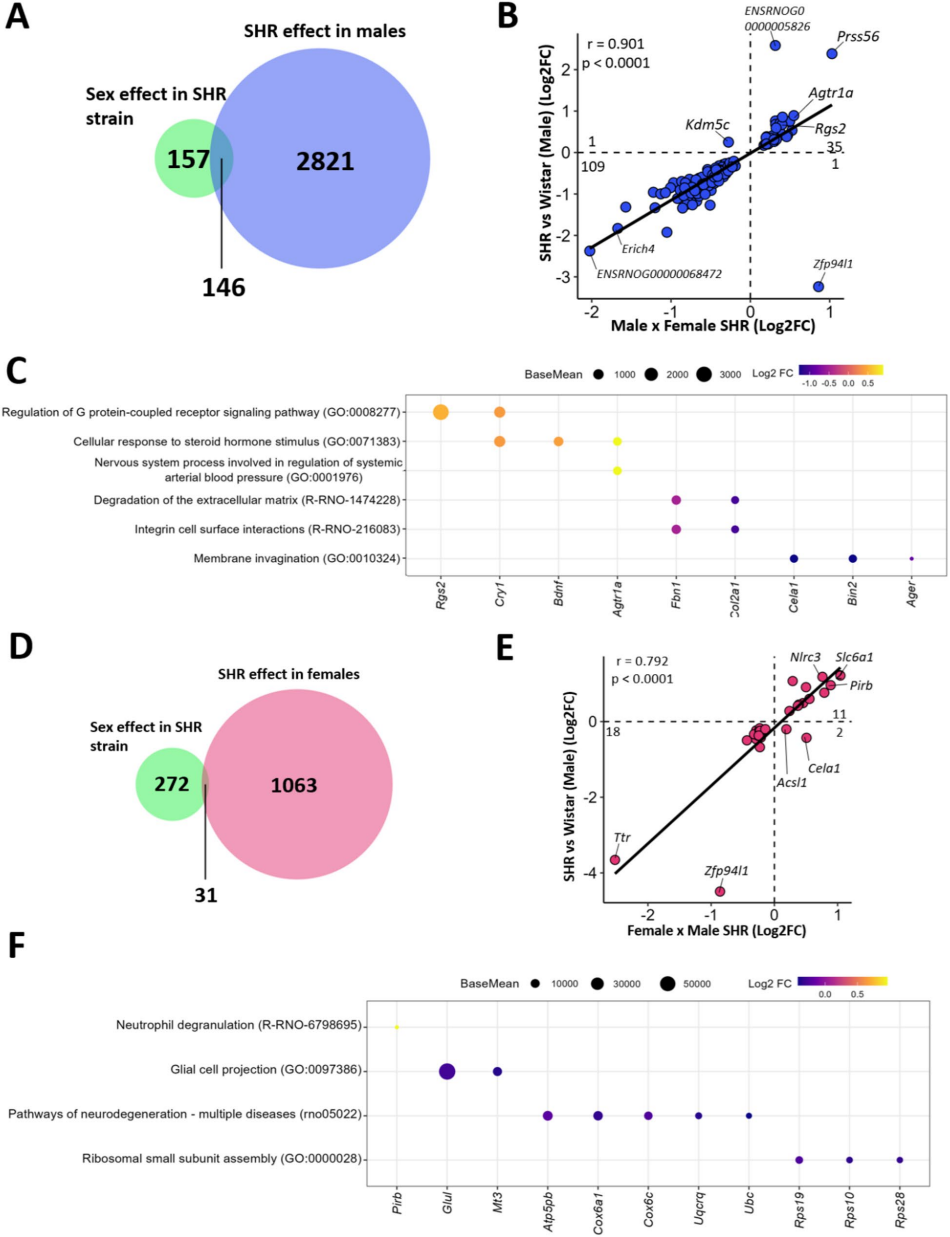
Sex Enrichment and Strain Differences: **(A)** Venn diagram showing the influence of sex on the coexpression of genes in SHRs (green) and the effects on SHRs compared with those in Wistar rats in males (blue). **(B)** Scatter plot showing the correlation (Spearman) between genes affected by sex in SHRs and those enriched in the SHR lineage compared with those enriched in the Wistar lineage in male rats, with the top 2 genes most affected in each direction highlighted, as well as two additional genes of interest. **(C)** Gene ontology analysis, demonstrating some of the pathways regulated between genes affected by sex in SHRs and those enriched in the SHR lineage compared with the Wistar lineage in male rats. The top five most highly expressed genes in each pathway are highlighted, with the size representing their mean base expression in males and the color indicating the log2-fold change in relation to the strain effect in males. **(D)** Venn diagram showing the influence of sex on the coexpression of genes in SHRs (green) and the effect of sex on female SHRs compared with Wistar rats (red). **(E)** Scatter plot showing the correlation (Spearman) between genes affected by sex in SHRs and those enriched in the SHR lineage compared with the Wistar lineage in female rats, with the top 2 genes most affected in each direction highlighted. **(F)** Gene ontology analysis, demonstrating some of the pathways regulated between genes affected by sex in SHRs and those enriched in the SHR lineage compared with the Wistar lineage in female rats. The top five most highly expressed genes in each pathway are highlighted, with the size representing their mean base expression in females and the color indicating the log2-fold change in relation to the strain effect in females

To identify key sex-specific drivers of hypertension, we correlated genes differentially expressed by strain with those differentially expressed by sex in the SHR groups. This approach revealed 146 genes commonly enriched in both the comparison between SHR and Wistar (SHR males versus Wistar male) and the comparison between sexes (Male SHR versus Female SHR) (Fig. 6A). A correlation analysis of these 146 genes revealed a strong and significant correlation (*r* = 0.901, *p* = 0.0001), with 35 genes being positively regulated in males and associated with the hypertensive strain, including *Adrenoceptor Alpha 2 A (Adra2a)*,* Angiotensin II Receptor Type 1 (Agtr1a)*,* Regulator Of G Protein Signaling 2 (Rgs2)*,* TNF Receptor Superfamily Member 12 A (Tnfrsf12a)*,* Atypical Chemokine Receptor 3 (Ackr3)*, and *Brain-Derived Neurotrophic Factor (Bdnf)* (Fig. 6B, Supplementary Table 14), neither of which was positively correlated with the *Androgen Receptor (Ar)* expression in male rats (Supplementary Table 15). The positively regulated genes were associated with steroid hormone receptor signaling (GO:0031958, GO:0071383, GO:0033143, GO:0030518, GO:0043401, and GO:0033145), nervous system processes involved in the regulation of systemic arterial BP (GO:0001976), and G protein regulation (GO:0045744, GO:0008277, GO:0001637, and GO:0004930). In contrast, the downregulated genes were associated with processes involving the extracellular matrix process (R-RNO-1474228 and R-RNO-216083) (Fig. 6C, Supplementary Table 16).

Moreover, in the female analysis, among the genes regulated by the hypertensive strain in females and those affected by sex in the SHR lineage, 36 genes that were commonly associated were identified (Fig. 6D), revealing a strong and significant correlation (*r* = 0.792, *p* = 0.0001). Among these genes, 11 were positively enriched in females and in the hypertensive strain; notably, they included *Nlrc3*,* Synapse Defective Rho GTPase Homolog 2 (Syde2)*, and *Paired Ig-like Receptor B (Pirb)* (Fig. 6E). Of these, only *Syde2* was positively correlated with *Estrogen Receptor 1 (Esr1)*, while no such correlation was observed with *Estrogen Receptor 2 (Esr2)* expression in female rats (Supplementary Table 17). Among the positively regulated genes, we observed specific pathways, such as neutrophil degranulation (R-RNO-6798695), whereas the negatively regulated genes influenced pathways associated with neurodegeneration (rno05022), the ribosomal complex (GO:0000028), and glial cell projection (GO:0097386) (Fig. 6F, Supplementary Table 18).

### Data integration uncovers conserved neuroinflammatory pathways across the brain

Alterations in genes related to neuroinflammation were also evident in our analysis of sequencing data from other brain regions involved in BP regulation, such as the NTS, CVLM, RVLM, where the authors compared female SHR rats with female normotensive Kyoto rats (12 and 16 weeks old) [58]. In analyses restricted to female rats, comparison between female SHR and female normotensive strains in the NTS, an important nucleus that inhibits and projects to the PVN, we identified 107 significant DEGs shared between the NTS and PVN, demonstrating a strong correlation among the common genes (*r* = 0.567, *p* < 0.001), which include genes involved in inflammatory pathways, such as the *Anxa1* and *Interferon Alpha Inducible Protein 27 (Ifi27) (Supplementary Fig. 3).* The common genes between the PVN and other key brain areas related to BP control also demonstrated a high correlation score in the RVLM (*r* = 0.545, *p* < 0.001) and CVLM (*r* = 0.491, *p* < 0.001), indicating a similar response shared across these tissues. Further integration of our PVN results with those from the three other regions (NTS, RVLM, and CVLM) revealed 62 genes shared across these comparisons *(Supplementary Fig. 3)*. Among them, the upregulation of Solute Carrier Family 11 Member 1 (*Slc11a1*) and downregulation of *S100 Calcium Binding Protein B (S100b)* emerged (Supplementary Table 19).

## Discussion

This study provides, for the first time, a comprehensive transcriptomic catalog of the PVN in male and female normotensive and hypertensive rats, revealing profound sexual dimorphism in the molecular pathways involved in the development of hypertension. Our findings demonstrate that, in males, hypertension is associated with a transcriptomic signature reflecting altered autonomic regulation and neuroinflammation, whereas females exhibit a distinct profile suggestive of protective, anti-inflammatory adaptations. These molecular data align with our physiological findings of heightened vasomotor/neurohumoral autonomic variability in SHR males compared with females; the SHR female autonomic profile is indicative of better prognosis and supports the finding of the PVN transcriptome.

Our autonomic analysis revealed significant cardiovascular differences driven by both hypertensive strain and sex, providing a physiological foundation for our transcriptomic findings. As anticipated, SHR of both sexes had significantly elevated BP and increased BP variability across all frequency bands (TV-BP, VLF-BP, LF-BP, and HF-BP) compared with their normotensive Wistar counterparts. The increase in BP variability in SHRs compared with Wistar has been described previously [59–63]. However, other authors found no evidence to support increased BP variability in SHRs [64]. This difference in findings may be attributed to the different ages of the SHRs used in the different experiments. It is acknowledged that older SHRs often develop arterial stiffening, which can mask or even attenuate these fluctuations [60, 65–67]. The young 14-week-old rats used in our study were in a phase of established but not advanced hypertension. Therefore, the observed increases in VLF-BP and LF-BP variability suggest heightened activation of both the Renin-Angiotensin System (RAS) and sympathetic outflow directed to the vasculature [38, 68, 69].

We also found increased HF-BP variability in SHRs. Unlike HF-HR which is entirely neurogenic (vagal) and complex by nature (involving stretch pulmonary, stretch cardiac reflexes as well as the baroreflex) [70, 71]. The HF-BP is purely mechanical created by inspiration-induced distension (by negative intrathoracic pressure) of great thoracic veins which lowers heart filling, stroke volume and BP by a few mmHg. In expiration the recoiling of thorax and veins restores heart filling, stroke volume and BP. This creates the oscillation of blood pressure at respiratory rhythm [70, 71]. When the thoracic veins are unloaded (hemorrhage, vasodilatation), inspiration-induced drop of heart filling, stroke volume and BP is greater [72], and this increases the HF-BP oscillation [38, 73–75]. Another modifying factor of HF-BP amplitude is the breathing pattern. Slow and deep breathing creates more negative pressure upon thoracic veins than fast and shallow breathing [70]. In our experiment, SHR did not exhibit changes in breathing frequency, as judged by the position of the HF peak in both the SBP and HR spectra (data not shown). It is possible that the somewhat smaller circulating volume in SHRs than in Wistar [76] could be reflected in the higher HF-SBP in SHRs.

Interestingly, the LF/HF HR ratio was lower in SHRs of both sexes than in Wistar rats. This suggests the activation of compensatory cardiac vagal mechanisms that counteract chronic BP elevation in the early phase of hypertension when BRS is still preserved in 14-week-old SHRs. In advanced stages of hypertension, the BRS decreases and predicts hypertension complications [77]. However, others identified reduction of BRS in SHR before 20 weeks-old rats using the pharmacological method[78]. They report no defect of the sympathetic branch of the baroreflex, only of the vagal branch which exhibited a reduction of the maximal response and of its gain. We applied the method of sequences where all spontaneously and simultaneously increasing SBP and PI pairs (vagal response) or decreasing SBP and PI pairs (sympathetic response) around the set point are identified and plotted. The mean slope of all curves denotes the sensitivity of both vagal and sympathetic part of the BRS.

Unexpectedly we observed higher LF-SBP variability in normotensive female Wistar in respect to males and could be assigned to increased spontaneous motor activity during the registration period. This finding is not so surprising and was observed by others in normotensive rats [79]. As concluded by Reckelhoff [80], there is no convincing evidence such as satisfactorily long-term blood pressure recordings to substantiate that gender-associated differences in BP observed in humans and hypertensive animals stand for normotensive rats, too.

Possibly due to the short-lasting recording of BP in our study and increased spontaneous motor activity during the registration period, SHR females did neither exhibit lower BP nor LF-BP variability in respect to SHR males. The LF-BP variability is a marker of sympathetic activity and has been shown to increase significantly under acute stressful conditions such as mental stress [81] hemorrhage [73], orthostatic challenge [82], and especially when the arterial baroreceptor reflex exhibits a resonance frequency close to the frequency of spontaneously occurring LF at 0.4 Hz [83]. However, under chronic conditions such as chronic hypertension, the increase of LF is not obvious and can be masked by vascular remodeling impairing vascular oscillatory performance [84].

A crucial finding indicating marked sexual dimorphism within the hypertensive group is the difference between total BP variability and VLF-BP variability, with SHR males exhibiting higher levels than SHR females. The VLF-SBP variability is a dominant component of total BP variability (> 80%). It is created by spontaneous vascular myogenic activity and modulated by the sympathetic nerves, hormones and locally generated vasoactive molecules [38]. The VLF-SBP component is almost abolished by α−1 adrenergic blockade or ganglionic blockade [69, 85] and restored after catecholamine infusions [38, 85]. Increased sympathetic activity (due to the dense innervation of macula dense by sympathetic nerves acting at β1 adrenergic receptors) triggers renin release which increases angiotensin II production. Angiotensin II then acts back to increase noradrenaline release at the periphery and enhance sympathetic outflow centrally, all involving AT1 receptors [86, 87], including vasopressin release [13]. Using pharmacological tools RAS was shown to contribute to VLF-BP variability [73, 88, 89]. Finally, elevated sympathetic tone and activation of the renin-angiotensin system are the pathophysiologic hallmarks of hypertension, and the interactions between these systems are particularly deleterious. The greater activation of the RAS in male SHR has already been demonstrated in the literature, with males showing higher involvement of the classical RAS pathway, which includes the conversion of angiotensin I (Ang I) to angiotensin II (Ang II) by angiotensin-converting enzyme (ACE) compared to females [90]. These differences observed in male SHR, indicates a distinct pattern of cardiovascular regulation in females, with the presence of differences mechanism, such as the well-documented estrogen-mediated protective effects on blood vessels and improved vascular compliance [91], upregulation of vascular endothelial β-adrenoceptor expression, which mediates vasodilation and reduces the reactivity of vasoconstrictors [92]. Furthermore, we observed that SHR females exhibited a higher HR compared to males, which may partially counterbalance the reduced TV-SBP and VLF-SBP components, contributing to maintain SBP levels like those observed in male SHR.

The sexual dimorphism in hypertension is well documented in humans, with men facing a greater risk than women do, particularly in early adulthood. Our transcriptomic analysis of the PVN revealed a greater number of DEGs in SHR males than in females, which may reflect sex-specific molecular differences associated with hypertension. PCA revealed that the samples clustered distinctly based on these two factors, with sexual dimorphism becoming dramatically more pronounced under hypertensive conditions. In the normotensive Wistar strain, the differences between sexes are modest, with DEGs associated with inflammasome activity in females, whereas in males, enrichment in the GABAergic, GPCR, and oxytocin signaling pathways is detected. However, in the hypertensive SHR strain, these differences were profound. The female hypertensive profile is characterized by changes in the extracellular matrix, whereas the male profile is dominated by a massive upregulation of pathways central to BP regulation, protein folding and secretion, as well as the emergence of pathways related to neurodegeneration, a condition strongly correlated with hypertension in the literature [93, 94]. These divergent molecular signatures strongly suggest that the central mechanisms driving hypertension in the PVN are fundamentally different between males and females.

The transcriptomic disruption in male SHRs is characterized by a clear prohypertensive signature, including key genes, such as the upregulation of the endogenous peptides *Hcrt* and *Bdnf*. These genes are highly relevant in the PVN, as both are implicated in the autonomic and neurohumoral regulation of BP [95–98]. This finding is further amplified by the increased expression of the critical GPCR *Agtr1a* (the Angiotensin II Type 1a receptor). The expression of *Agtr1a* has been shown to be regulated by *Bdnf *[97], as well as to BP regulation by reducing inhibitory GABAergic input and increasing excitatory glutamatergic input to PVN neurons, thereby enhancing excitatory autonomic drive [99–102]. In contrast to its role in BP control, the association of AGTR1 gene variants in humans with hypertension remains debated, as the evidence from population studies is still inconclusive [103]. However, the upregulation of these genes in male SHR provides a direct molecular explanation for the increased of VLF-SBP and LF-SBP (hallmarks of sympathetic and RAS activation) observed in comparation with the normotensive strain. This prohypertensive state is also compounded by neuroinflammation [104, 105], as evidenced by the increased expression of genes such as the GPCR *Ackr3* [106]. The broad upregulation of pathways related to protein transcription, folding, and cellular stress likely reflects the high metabolic demand placed on PVN neurons by this state of chronic sympathoexcitation and neuroinflammation [78, 79].

In contrast to males, females presented a smaller change in the transcriptomic profile within their PVN, which was more constrained and pointed toward a protective, anti-inflammatory response. Among the DEGs, the downregulated gene *Anxa1* plays important roles in the functionality of the nucleus because of its involvement in preventing neuroinflammation [107]. The decrease in the expression of this gene is consequently associated with a proinflammatory state. Most notably, we observed the coordinated regulation of key inflammasome components: the upregulation of the catalytic receptor *Nlrc3*,* which is* known to inhibit inflammasome activity [108–110], and the concurrent downregulation of *Aim2*, a sensor protein that initiates inflammasome assembly [111]. In addition, we observed downregulation of the inflammatory gene Ifi27 [112] in both the PVN and NTS, whereas the inflammatory gene S100B [113] was downregulated in all four regions analyzed: PVN, NTS, RVLM, and CVLM. The simultaneous upregulation of an inhibitor and downregulation of an activator suggests a concerted, multipronged mechanism in females designed to actively suppress neuroinflammation in the PVN, as well as in other key brain regions involved in BP control. The higher participation of anti-inflammatory components in SHR females is also observed in the kidneys, where there is a higher prevalence of anti-inflammatory T regulatory (Treg) cell infiltration compared to males, who present a higher infiltration of pro-inflammatory Th17 cells [114]. This active immunomodulation observed in the PVN, likely explains pathways related to the neuronal ensheathment, myelination, and glial cell differentiation observed in females. We propose that compensatory anti-inflammatory mechanisms are key factors underlying the distinct hypertensive phenotype observed in females, as reflected by their lower total and low-frequency SBP variability compared with males.

Despite the profound sex-specific differences, 555 genes were commonly regulated by hypertension in both sexes, revealing a core pathological response. These shared genes constituted a smaller fraction of the total DEGs in males but accounted for more than half of the changes in females, underscoring the highly distinct mechanisms driving hypertension in males. Among these common genes, there was remarkable conservation in the direction of alteration between sexes, as demonstrated by the strong correlation in their expression. However, females presented a higher coordinated transcriptomic response to hypertension than did males within this shared gene set. Central to this shared signature is a proinflammatory state driven by the downregulation of Anxa*1*, this gene encoding an important anti-inflammatory peptide [107], which is reduced in both sexes and in female NTS, driving a proinflammatory basal state likely contributing to neuroinflammation, an important condition in PVN already associated with hypertension [115]. Alongside, we observed the common upregulation of *Slc11a1* in both sexes and across the NTS, RVLM, and CVLM, a pro-inflammatory gene previously linked to microglial activation and neuroinflammatory processes [116]. The inflammation findings are supported by evidence of glial dysfunction, as indicated by the downregulation of *Aqp4*, an ion channel crucial for astrocyte functionality in the hypothalamus, indicating dysfunction in this type of cell functionality [117]. Another important upregulated common gene was *Sgk1*, an enzyme-linked to cellular stress and death, which indicates a negative feedback mechanism involving neurotrophic factors such as BDNF, leading to decreased neuronal survival and function [118]. Together, these findings suggest that hypertension establishes a common foundation of neuroinflammation and glial dysfunction in the PVN, upon which more dramatic and sex-specific mechanisms are built.

To better understand the sex-specific impact of the PVN in driving hypertension, we correlated the genes differentially regulated by sex in SHRs with those influenced by hypertension. This analysis effectively isolated distinct molecular drivers underlying the divergent trajectories observed between males and females. In males, this approach highlighted genes such as *Agtr1a* and Rgs2 as central to the hypertensive state (upregulated in SHRs compared with Wistar) and male-enriched (upregulated in SHR males compared with SHR females). Recent findings have shown that the overexpression of *Rgs2* in the PVN is associated with the attenuation of BP increases and sympathetic outflow caused by angiotensin II and its receptor, likely acting as a compensatory mechanism to control hypertension [119]. Notably, variants in RGS2 in humans have been associated with hypertension in genetic studies [119], underscoring its conserved role across species in BP regulation. The differential regulation of *Rgs2* and *Agtr1a* potentially affects the mechanism of the RAS pathway, suggesting a possible mechanism underlying the increase in VLF-SBP observed in SHR males compared to females. Additionally, a positive correlation between the gene expression of males and the SHR strain was observed in the expression of *Bdnf*, encoding a peptide involved in the activation of sympathetic tone [96]. Although these genes were enriched in males and in the hypertensive strain, none was found to be correlated with androgen receptor expression, which suggests that these key genes are probably not directly linked to androgen receptor mediated mechanisms. On the other hand, comparison of genes enriched in females and in the SHR strain revealed several interesting candidates. Among these, *Syde2*, a gene related to synapse development and plasticity emerged as a key gene also correlated with *Esr1*, suggesting a female-specific mechanism possibly modulated by estrogen. We also identified increased expression of *Nlrc3* and *Pirb*, both involved in anti-inflammatory pathways [108–110, 120]. These findings suggest that these anti-inflammatory genes are possible key drivers of the protective female phenotype, likely acting as compensatory mechanisms to reduce neuroinflammation, which is a hallmark of the hypertensive PVN.

While this study provides a comprehensive transcriptomic overview, some limitations must be acknowledged. The drawback of our study is the lack of 24 h-long BP measurements which would ascertain better assessment of mean values of BP and HR and differences between strain and sex. However, the phenomenon we analyzed short-term cardiovascular variability reflects autonomic cardiovascular control. It occurs in seconds and less and one-hour BP recording period was largely ample to allow time-spectral analysis which additionally considered variations in time of each spectral component. To minimize the effect of longer-term variations of BP (ultradian and circadian) on the amplitude on short-term variability we applied linear detrending of BP signal before analysis. It is well acknowledged that in humans, sympathetic control of BP exhibits greater influence on BP during the day. However, unlike in humans whose wake-sleep pattern is circadian (24 h) and monophasic (awake during day and sleep over night), in rats it is ultradian and polyphasic (multiple short naps throughout the day and night) [121]. Therefore, the translation of rats to human sleeping cycle is not straightforward, especially regarding autonomic BP control.

Another key point is that we used bulk RNA sequencing, which allowed us to obtain a general overview of the transcriptomic effects of sex and hypertension in the PVN. Although similar group sizes have been successfully used in transcriptomic studies of other key brain regions involved in blood pressure control in SHR animals, enabling efficient and statistically robust analyses [58, 122, 123], this relatively small sample may still limit the detection of more subtle transcriptional changes and, together with the bulk approach, prevents the attribution of these effects to specific cell populations. The limitations related to the lack of cellular resolution represent an important gap in the PVN, given its high cellular complexity (e.g., astrocytes, and microglia) and diversity of neuronal types (e.g., magnocellular neurons, and PCN*)*. An additional key limitation is due to the absence of proteomic information. Due to the lack of proteomic measurements, it is not possible to assess the translation of RNA into protein or the activity of these proteins in the regulation of BP and neuroinflammation. Finally, another important limitation is that our findings regarding DEGs cannot determine whether the observed transcriptomic changes are a cause or a consequence of hypertension. Further experimental manipulation or pharmacological modulation will be necessary to clearly identify the roles of some key genes identified, as well as to assess their involvement at different developmental stages (e.g., juvenile, adult, and aged animals).

In summary, our results reveal sexual dimorphism in the transcriptomic landscape of the PVN in hypertension supporting hemodynamic data pointing to lower VLF-BP variability in SHR females suggesting less synergism of sympathetic nervous system and the RAS, hallmarks of deleterious hypertension. That is molecularly substantiated by the male-exclusive upregulation of prohypertensive genes such as *Bdnf*,* Hcrt*, and the RAS component *Agtr1a*. In addition to these prohypertensive genes, the upregulation of *Rgs2* in males, compared with females, suggests a compensatory mechanism to oppose the angiotensin II cascade. It was possible to observe an inflammatory state in both sexes, mainly through the downregulation of Anxa1 and the upregulation of Slc11a1, demonstrating a common inflammatory status across brain regions involved in BP control. In contrast, SHR females displayed gene expression patterns related to anti-inflammatory pathways, such as the downregulation of *Aim2*, *S100b*, and *Ifi27*, and the upregulation of *Nlrc3* and *Pirb*, being these changes observed exclusively in females. This molecular profile may help explain the lower prevalence and severity of hypertension in females, as well as the differences in cardiovascular disease occurrence between sexes, likely driven by the active suppression of genes involved in inflammatory pathways and the absence of key genes regulating vasomotor/neurohumoral autonomic control observed in males.

## Conclusion

In conclusion, our analyses provide new insights into the cardiovascular and molecular correlates of sex differences in hypertension. We identified the presence of a neuroinflammatory mechanism in the PVN, suggesting a strong contribution to the hypertensive state. Our results also revealed a distinct neurohumoral autonomic profile in males, characterized by enhanced RAS-mediated sympathetic drive, whereas in females, a stronger anti-inflammatory component was observed, which may explain the lower prevalence and severity of hypertension in females. These findings offer a valuable framework for the development of future sex-specific pharmacotherapeutic strategies.

## Perspectives and significance

Our current study revealed several mechanisms contributing to the hypertensive state, as well as compensatory mechanisms across sexes. Our analyses provide new insights into the relationship between hypertension and PVN, and highlights mechanisms that could be targeted for BP control. Future transcriptomic studies will expand these analyses to the cellular level, for example, through single-cell RNA sequencing (scRNA-seq) offering a broader perspective on the role of the PVN in hypertension, as inferring through manipulation or pharmacological modulation some of the key genes identified in this study, as well as to assess their involvement at different developmental stages (e.g., juvenile, adult, and aged animals).

## Supplementary Information

Supplementary Material 1.

Supplementary Material 2

## Data Availability

In addition to the data reported in the manuscript and additional files, all the raw data files are available through the NCBI Gene Expression Omnibus (GEO) platform (GSE: GSE309435).
