# Supplementary material for: Sex-specific hypothalamic PVN transcriptomic signatures of blood pressure autonomic regulation and neuroinflammation in hypertension
Source: Biol Sex Differ. 2026 Feb 21;17:46. doi: 10.1186/s13293-026-00855-3 (PMC12964816; doi:10.1186/s13293-026-00855-3)
Supplement: Supplementary file 2 — Supplementary Material 2 [file 13293_2026_855_MOESM2_ESM.docx]

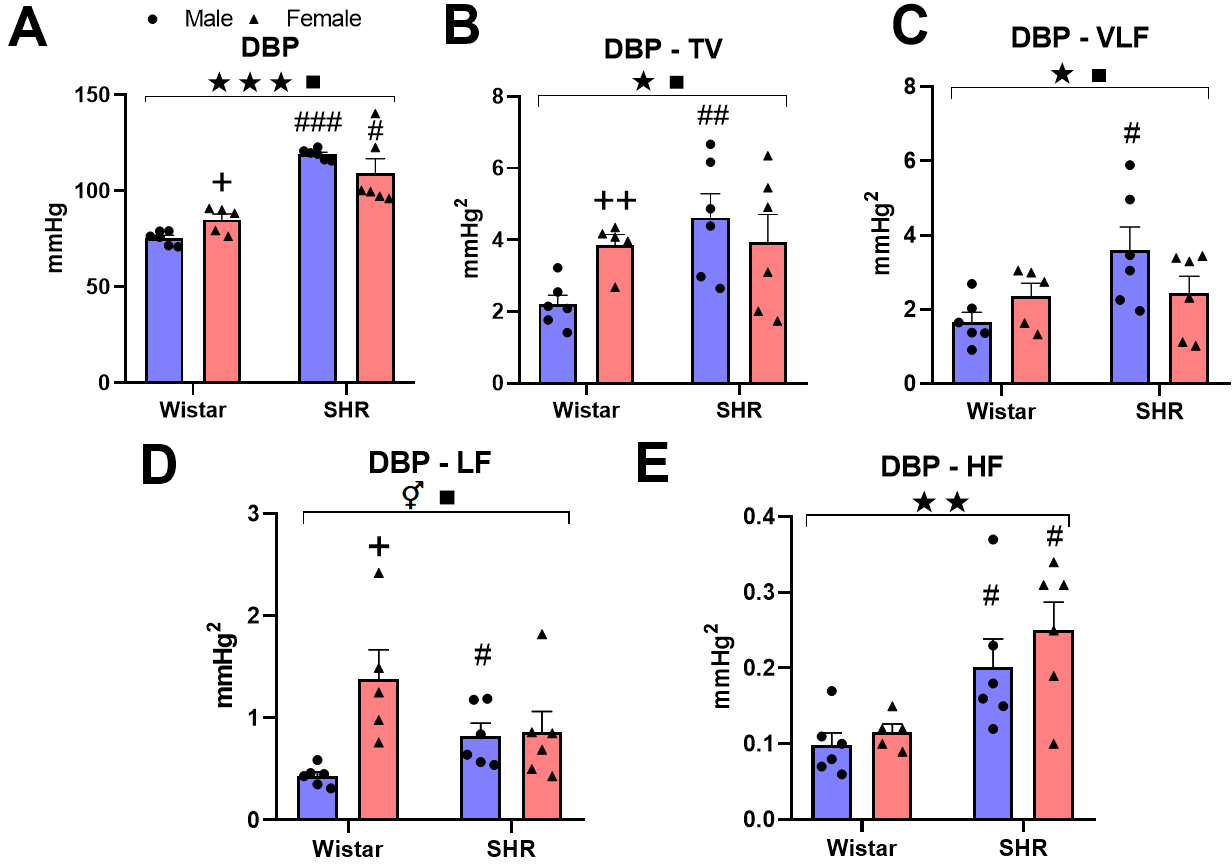


**Supplementary Figure 1 - Diastolic BP Levels and Variability**: **(A)** Bar plot showing DBP values across sex and lineage, as well as short-term DBP variability in SHR and Wistar of both sexes. The blue bars indicate males, whereas the red bars indicate females. (B–E) Bar plots representing short-term variability in total variance **(B)**, very-low-frequency (VLF) **(C)**, low-frequency (LF) **(D)**, and high-frequency (HF) **(E)** components. Note the effects of the strain and the strain × sex interaction on DBP and DBP variability. ★ p<0.05, ★★ p<0.01, ★★★ p<0.001: strain effect (comparison between SHR and Wistar rats within the same sex); ⚥ p < 0.05: sex effect; ∎ p < 0.05, ∎∎ p <0.01: strain × sex interaction (two-way ANOVA); + p<0.05, and ++p<0.01: female compared with male within the same strain; and #p<0.05, ##p<0.01 and ### p<0.001: intergroup comparison (comparison between SHR and Wistar rats within the same sex; Tuckey post hoc).


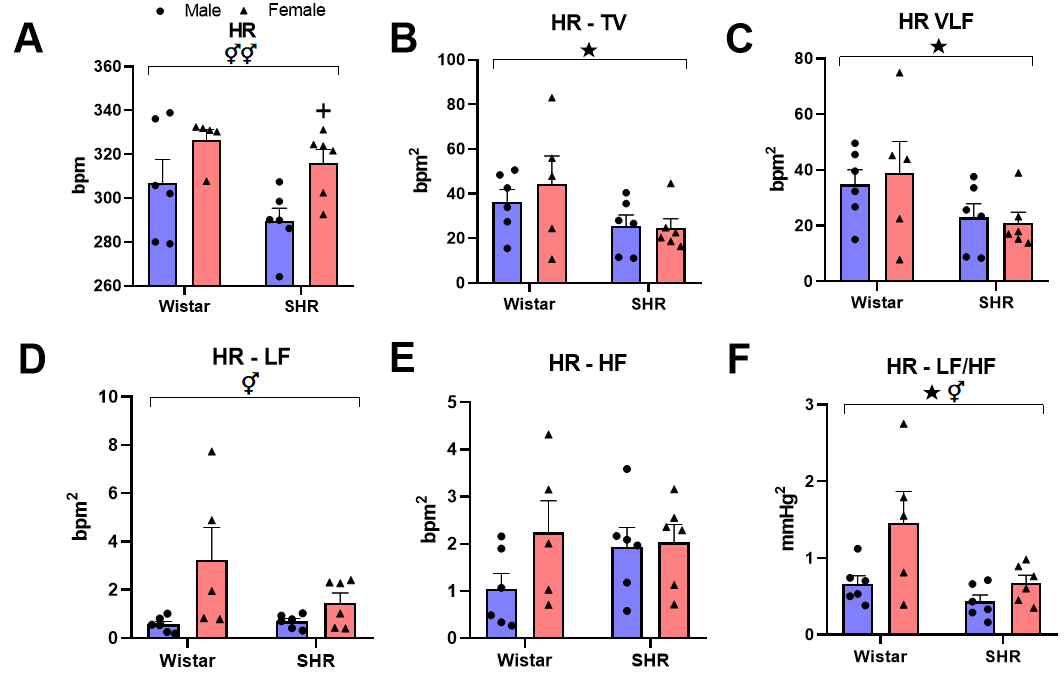


**Supplementary Figure 2 - Heart Rate and Variability**: **(A)** Bar plot showing heart rate (HR) values across sex and lineage, as well as short-term DBP variability in SHRs and Wistar of both sexes. The blue bars indicate males, whereas the red bars indicate females. (B–E) Bar plots representing short-term variability in total variance **(B)**, very-low-frequency (VLF) **(C)**, low-frequency (LF) **(D)**, and high-frequency (HF) **(E)** components and the LF/HF ratio **(F).** Note the effects of the strain and the strain x sex interaction on HR and HR variability. ★ p<0.05: strain effect (comparison between SHR and Wistar rats within the same sex); ⚥ p < 0.05, ⚥ ⚥ p < 0.01: sex effect (two-way ANOVA). + p<0.05: female compared with male within the same strain (Tuckey post hoc).


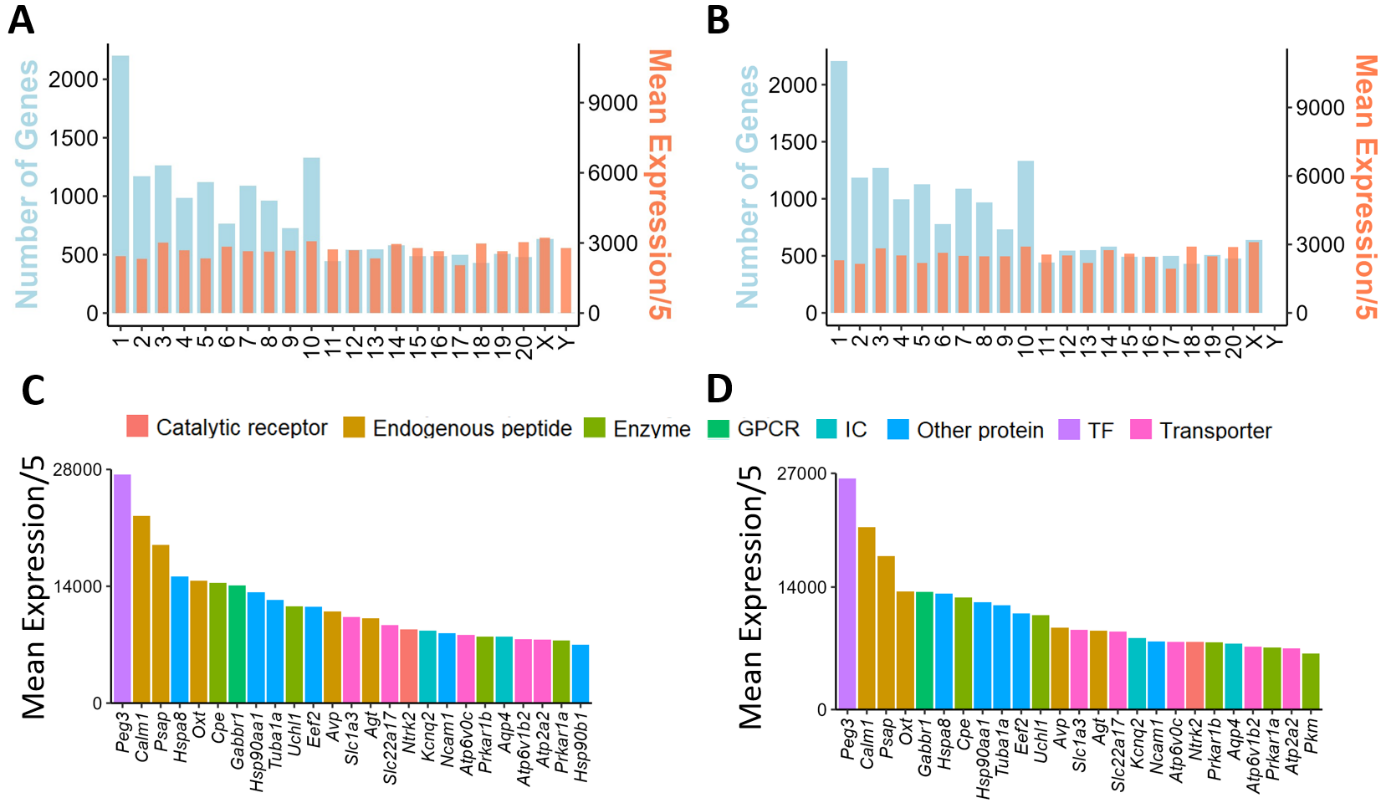


**Supplementary Figure 3 - Chromosomal and Tissue Gene Distribution by Sex**: Bar plot showing the number of genes in each chromosome (blue) and their mean gene expression (divided by 5) of the genes (orange) in male **(A)** and female **(B)** rats from both strains. Bar plot representing the mean expression (divided by 5) of the 25 genes most highly expressed in the PVN from both strains in males **(C)** and females **(D)**, categorized according to IUPHAR.


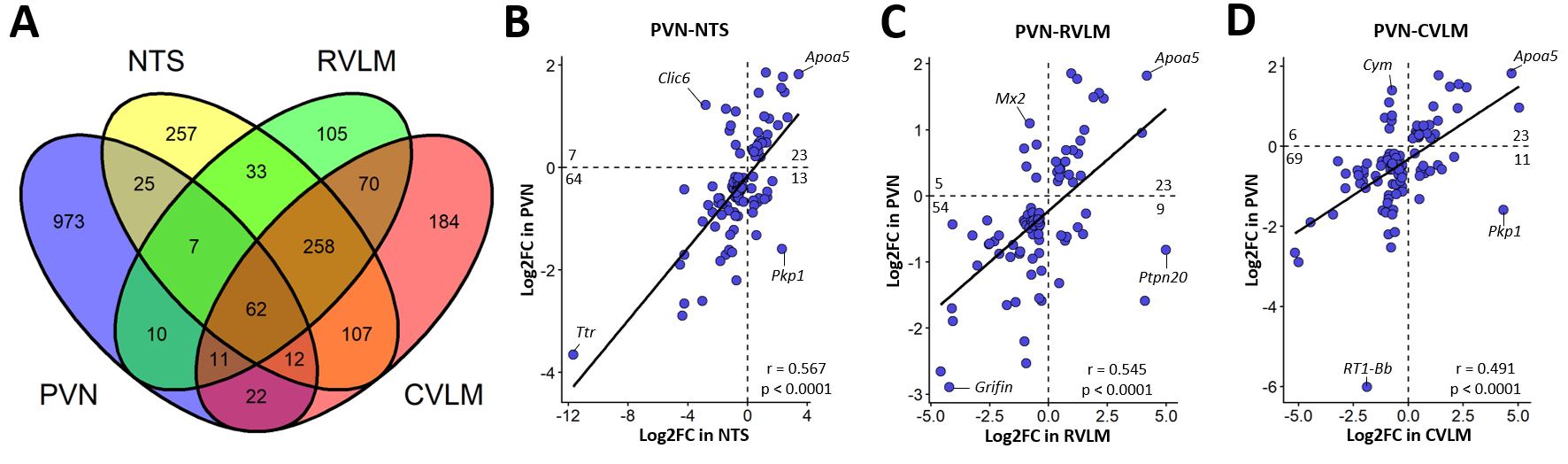


**Supplementary Figure 4 – Data Integration: (A)** Venn diagram demonstrating the number of common genes between the PVN (SHR female compared to female Wistar) and the NTS, RVLM, and CVLM (SHR female compared to female Wistar Kyoto). **(B–D)** Scatter plots showing the Spearman correlation among the genes shared by the PVN (SHR female compared to female Wistar) and each region: NTS **(B)**, RVLM **(C)**, and CVLM **(D)** (each: SHR female compared to female Wistar Kyoto). The most affected gene in each quadrant is labeled.
